# Supplementary material for: Who is absent and why? Factors affecting doctor absenteeism in Bangladesh
Source: PLOS Glob Public Health. 2024 Apr 4;4(4):e0003040. doi: 10.1371/journal.pgph.0003040 (PMC10994372; doi:10.1371/journal.pgph.0003040)
Supplement: Attachment — Submitted filename: Response to Reviewers_ACE Bangladesh.docx [file pgph.0003040.s002.docx]

**Point by point response to editor, journal and reviewer suggestions**

1. Please include a complete copy of PLOS’ questionnaire on inclusivity in global research in your revised manuscript.

We have attached a completed questionnaire.

2. Please send a completed 'Competing Interests' statement, including any COIs declared by your co-authors. If you have no competing interests to declare, please state "The authors have declared that no competing interests exist". Otherwise please declare all competing interests beginning with the statement "I have read the journal's policy and the authors of this manuscript have the following competing interests:"

We have added the declaration from the paper to the online system.

3. Please amend your detailed Financial Disclosure statement. This is published with the article. It must therefore be completed in full sentences and contain the exact wording you wish to be published.

a. State the initials, alongside each funding source, of each author to receive each grant.
b. State what role the funders took in the study. If the funders had no role in your study, please state: “The funders had no role in study design, data collection and analysis, decision to publish, or preparation of the manuscript.”
c. If any authors received a salary from any of your funders, please state which authors and which funders.

If you did not receive any funding for this study, please simply state: “The authors received no specific funding for this work.”

We have amended the financial disclosure to specify the contract was awarded to author MHK and that funders had no role in study design, data collection and analysis, decision to publish, or preparation of the manuscript.

4. Please provide separate figure files in .tif or .eps format only and remove any figures embedded in your manuscript file. Please also ensure all files are under our size limit of 10MB.

For more information about figure files please see our guidelines:

<https://journals.plos.org/globalpublichealth/s/figures> 
<https://journals.plos.org/globalpublichealth/s/figures#loc-file-requirement>

5. We have noticed that you have a list of Supporting Information legends in your manuscript. However, there are no corresponding files uploaded to the submission. Please upload them as separate files with the item type 'Supporting Information'.

We have added reference to the Strobe checklist in the manuscript and included a list of supporting documents after the references as requested.

6. Tables should not be uploaded as individual files. Please remove these files and include the Tables in your manuscript file as editable, cell-based objects. For more information about how to format tables, see our guidelines:

<https://journals.plos.org/globalpublichealth/s/tables>

We have incorporated tables into the main manuscript.

Reviewer #1: This manuscript is based on research on an important topic and deserves publication. However, the authors need to address some issues, especially the discussion section requires significant revision.

**Abstract**:
“Respondents who did not serve….”: The meaning of the sentence is not clear. Please revise.

Response to Reviewer: Thank you for your feedback. We have revised this sentence for clarity as follows:

*Respondents who did not serve their entire rural posting were less likely to report an unmanageable workload than respondents who did (AOR= 0.39, 95% CI 0.22-0.70).*

**Introduction:**
What does ‘regional communities’ mean in the second paragraph? Does it mean ‘rural communities’?

Response to Reviewer: Yes we were referring to rural communities – we have revised this sentence as suggested:

*Doctor absenteeism in Bangladesh disproportionately affects rural communities who already experience reduced access to care relative to their urban counterparts*

“An increasing body … all doctors equally”: The meaning of the sentence is not clear. Please revise.

Response to Reviewer: We have simplified this sentence to help clarify its meaning.

Specifically: ‘*An increasing body of evidence has argued that this could be a result of the failure of such measures to address the key incentives driving the behaviour of doctors and account for the different contexts they face.*

“Instead, we argue … across the system”: Please revise to simplify the sentence.

Response to Reviewer: This line is now simplified.

*Instead, we argue that interventions targeting the key drivers of doctor absenteeism offer the greatest promise for reducing absenteeism across the system*

“Depending on the degree … periods without sanction”: Please provide a reference. The same reference from Joarder et al. (2018) that was used before demonstrated that doctors' political affiliation contributed to their avoidance of the 2-year compulsory rural service.
Any reference will suffice, however.

Response to Reviewer: We have added the following references in support of this point.

Odii, Aloysius, et al. "Absenteeism in primary health centres in Nigeria: leveraging power, politics and kinship." *BMJ global health* 7.12 (2022): e010542.

Joarder, Taufique, et al. "Retaining doctors in rural Bangladesh: a policy analysis." *International Journal of Health Policy and Management* 7.9 (2018): 847.

Also, what does sanction mean in this sentence? Please revise for clarity.
Response to Reviewer: We have revised this for further clarification.

*Depending on their level of political connections, doctors reported varying degrees of absenteeism: those with influence, power and access to networks could be absent from their duty places for long periods often without consequence*

**Survey design:**
‘or the impact of sanctions’: What does this mean?

Response to Reviewer: This is now removed for the sake of clarity.

“All doctors in Bangladesh … left this post early”: Not following their training; following their recruitment in the public sector. Please revise accordingly.

Response to Reviewer: This line is now revised. See:

*All doctors in Bangladesh are required to serve two years in a rural facility following their recruitment in the public sector, and we defined absenteeism as respondents reporting that they left this post early*

**Analysis:**
Please provide a reference to the software Stata (Version 13.0).
Response to Reviewer: Added a reference for Stata (Version 13.0).

**General characteristics:**
“Table 1 outlines …: To align with the table, please report the age first, then gender, and other variables as per the sequence they are listed in the table.

Response to Reviewer: This line is now revised accordingly.

Delete ‘(Table 5)’ from that sentence. It is incorrect.

Response to Reviewer: Deleted. Thanks.

“At present, 47% …”: No need to mention all categories in the text unless it imparts any important message. Please delete from 20% onwards.

Response to Reviewer: This line is now revised and only medical officer is reported.

Experiences at the rural facilities:
‘Almost all respondents’: Mention 96% within parenthesis.
Response to Reviewer: Revised accordingly.

‘transferred to sanctioned posts’: Transferred or posted. Please replace it with 'posted' if that is correct.
Response to Reviewer: This is now revised. Thanks.

“About one third of the respondents…”: Instead of writing that 37% stayed in the residence, please write that more than three fourth of the respondent did not stay in the residences provided to them on campus. This sounds more intriguing.
Response to Reviewer: We have rephrased this point in line with your suggestion. The 76% statistic in Table 2 refers to families staying in the residence with respondents, we have incorporated this point here too. See:

*Almost two-thirds (63%) of the respondents did not stay in the residence provided by the authority most of the time and over three-quarters (76%) of respondent’ families did not stay in the residence provided*

‘lack of basic amenities (73%)’: Wrong. It is 72%, according to Table 2.

Response to Reviewer: Yes. It is now corrected.

“A significantly greater proportion …”: Unclear sentence. Please revise.
Response to Reviewer: This sentence is now revised for better understanding. See:

*A significantly greater proportion of doctors (82%) who served their rural post uninterruptedly reported that the workload in the rural facilities was not manageable and around 67% of the doctors who served their rural post uninterruptedly thought the workload was manageable (p=0.004) (Table 3).*

“Interestingly, no significant … “: Meaning not clear. Also, the significance of this finding is not clear. Please delete it if not important.
Response to Reviewer: This line is now deleted.

**Factors associated with absenteeism:**
Mention Table 5, where these findings are documented.

Response to Reviewer: We have mentioned table 5 in the first line of the section.

“Three models were considered …”: I am not sure why the authors need three different models to convey the same message or findings, which they can easily do with one inclusive model (model iii). If there is no important reason, please revise this part (and the methods, too) to show only one model, including all variables.
Response to Reviewer: We have used three models to investigate the impact of of networking (I) and work experience (II). We have added a justification for this in the paper:

*For the final regression model, three multiple logistic regression analyses were performed to investigate the impact of individual socio-demographic characteristics, work experience and networking to a doctor serving out their full rural posting.*

“In model I, networking … “: In Table 5, this set of variables was termed as 'factors favorable for career progression'. Please use the correct term consistently and ensure the terms used are aligned between the text and the table.

Response to Reviewer: We have termed the model I as networking and where we have used only the factors ‘favorable for career progression’; model II as work experience where we have used the factors related to work experience from rural facility and finally the model III as full model where we have used both of the factors. We have now revised the interpretation to make it reader friendly. Thanks for your suggestion.

AOR = 0.016: This is wrong. The AOR is 2.15. This is the p-value. Please revise.
Response to Reviewer: This number is now revised. Thanks.

“In the final … absenteeism.”: Absenteeism or uninterrupted service? These variables are different, so please clarify.
Response to Reviewer: Changed to uninterrupted service.

**Discussion and conclusions:**
Paragraph 1: This paragraph is redundant. The first sentence may be used in the background section, where the authors try to demonstrate the importance of the research topic. This is unnecessary in the discussion section as the importance or significance of the research is supposed to be established already.

Response to Reviewer: We have restructured the discussion extensively in line with these comments.

‘facing doctors’: Instead, use ‘doctors face’.
Response to Reviewer: Revised accordingly.

“These include but … “: New findings are introduced in the discussion which are unsupported by data.
Response to Reviewer: This line is now revised, and all the findings are supported by our survey (Table 2 and 3).

‘intelligent use’: Please avoid loaded phrases like this and report what you found exactly.
Response to Reviewer: This line is now revised.

‘leave vacancies’: What is this?
Response to Reviewer: We have changed to vacancies.

“However, this study … to be significant”: Wonder why. Any explanation?

Response to Reviewer: This might be because of the limited sample size and only a few of them were involved in private practice (n=91), lived in the residence (n=166), and family stayed at rural posting (n=40).

“While attention has … present at work”: Not sure how do the authors draw such a conclusion when their research does not include the former variables to compare?
Response to Reviewer: We have restructured the discussion extensively in line with these comments.

‘paying attention to … provision of logistics’: How does the data support this?

Response to Reviewer: We have restructured the discussion extensively in line with these comments.

‘pre-planning to ensure … transparent career path’: How is this found in the research?

Response to Reviewer: We have restructured the discussion extensively in line with these comments.

“When these are combined … absenteeism of doctors”: How do the authors know that? Any evidence?

Response to Reviewer: We have restructured the discussion extensively in line with these comments.

“Besides, WHO … at the earliest”: How is this related to the study?

Response to Reviewer: We have restructured the discussion extensively in line with these comments.

The last paragraph: Not supported by the data presented in the manuscript.
Response to Reviewer: We have restructured the discussion extensively in line with these comments.

**Major concerns:** Overall, the discussion and conclusion sections need to be overhauled.
In the discussion section:
1. State the study's major findings in the first paragraph in a direct, declarative and succinct manner. You may select two or, at best, three major findings from your study.
2. Write one paragraph or two for each major finding by explaining the meaning and importance of the findings.
3. Relate the findings to those of similar findings, preferably from Bangladesh or similar settings.
4. Mention the limitation and strengths of the study. This is somehow addressed already.
5. Provide your opinion about future research and/or policy implications, research/policy recommendations.

In the conclusion section or the last paragraph of the discussion section, give a take-home message, i.e., a couple of sentences summarizing your research and things you want the reader to remember, at the least from your article.

The current discussion section suffers from some serious flaws, such as over-interpretation of results, speculations, tangential issues, and, most importantly, the introduction of new issues that are not presented in the results and proposing conclusions or recommendations unsupported by data.

Response to Reviewer: We have restructured the discussion extensively in line with these comments.

**Reviewer #2:** This paper is straightforward and clearly written. I have a few ovearching comments and several more minor comments that the authors might consider in order to make the analysis sharper.

First, the finding that respondents who did not serve the entire rural posting were less likely to report an unmanageable workload is somewhat counterintuitive. The authors could point this out (somone reading quickly might even understand the reverse) and explore why. For example, perhaps simply saying made one interpret the workload as more, or, there was a greater liklihood among those willing to take on a higher workload to also stay.

Response to Reviewer: This line is now revised (Section 3.2) and we have discussed the importance of this result in greater detail in the revised discussion. For example, see:

*Interestingly, those who reported an unmanageable workload were found to be less likely to report leaving early. While this may at first seem counterintuitive, the finding aligns with other emerging literature that has highlighted the importance of social and political networks for doctors in securing preferred urban postings; those without such networks had no other alternative than to continue despite heavy workload. Taken together, our findings highlight the importance of the contextual factors in determining rural absenteeism and provide further evidence as to why traditional top-down approaches to doctor absenteeism have so far been ineffective.*

Second, the study explores both unsupportive or threatening behavior from caregivers and community members as distinct from political connections. What is the definition of political connections? How was this question asked? Is it to local politicians or higher up? Are these two constructs related conceptually in any way? (this isn't a measurement question so much as a question of your readers' ability to understand the findings).
Response to Reviewer: In our study, 'political connections' refer to any affiliations, associations, or relationships that individuals may have with local politicians or higher-level government officials. These connections could encompass personal relationships, affiliations with political parties or interest groups, or any form of interaction or influence that individuals have with those in positions of political power.

The introduction defines absenteeism as unscheduled work absence, but the study seems to focus on leaving the post early (rather than disappearing to Dhaka for a month for training, for example). The exact definition used and to what extent this tracks with the broader literature should be explained.
Response to Reviewer: Thanks for your comment. The introduction section in this manuscript cites the more frequently used definition of absenteeism which refers to loss of schedule time due to unscheduled work and how it is affecting the Bangladeshi health system. However, this paper focuses on the past experience of physicians on uninterrupted service provision with regards to absenteeism during the mandatory two years rural positions. The findings reflects about the factors and associations with regards to the uninterrupted service provision during their rural placements. We have clarified this in the paper:

*Specifically we defined absenteeism in our analysis as leaving a rural posting early, for any reason.*

The Introduction cites an estimate from the MoHFW regarding absenteeism. Is this 58.7% figure for any given day? (ie if I were to show up at every facility in the country on the same day, 58.7% of the HCWs would not be present). This is somewhat different from your leaving the post early definition.

Response to Reviewer: Anti-Corruption Commission (ACC) the anti-graft watchdog conducted a drive in 11 government hospitals and health centres in eight districts, including Dhaka. About 40% of doctors in 11 public hospitals and health centres were found to be absent in workplace during a drive conducted by the Anti-Corruption Commission (ACC).^[1]^ The 58.7% is for any given day.

The introduction refers to failed regulatory approaches the government has taken. It would be helpful to the reader to add a few words explaining what some of these have been.

Response to Reviewer: All sub-district and district level government hospitals of Bangladesh installed office attendance monitoring system using biometric fingerprint detection during 2012^[2]^. The date and time of arrival to and leaving the office of the officers and staffs are recorded in the system and allow reading from the head quarter.

Despite the regulatory attempt, absenteeism continued. An Anti-corruption drive in 2019 reported 81 out of 131 doctors were absent in eight hospitals outside the capital city Dhaka, with an absent rate of 61.8%^[3]^.

The administrative complications make the disciplinary actions time-consuming. When doctors remain absent, the authorities in most cases take three to six months to replace them. When a doctor remains absent for 30 consecutive days without permission, the facility chief is obligated to report the matter to higher authorities, health officials said. In many cases, however, the facility authorities do not report timely about the absent officer^[4]^.

We have revised the text to reflect these regulatory approaches.

The introduction also notes that interventions to overcome absenteeism do not impact all doctors equally. I understand what you mean, but perhaps I assume that your point is not about equality. Rather, it is about the extent to which these interventions meaningfully shift incentives in different contexts?

Response to Reviewer: Thanks for the clarification. You rightly mentioned that the point here is not about equality rather about designing interventions to meaningfully shift incentives based on context. We have revised the main text.

The section on survey design should cite the qualitative work (presumably the 2015 study).

Response to Reviewer: We have added the following reference from our qualitative work.

Absenteeism among doctors in the Bangladesh health system: What are the structural drivers? Nahitun Naher ^a^, Dina Balabanova ^b^, Martin McKee ^b^ ^d^, Mushtaq H. Khan ^c^, Pallavi Roy ^c^, Syed Masud Ahmed ^a^, Eleanor Hutchinson . [SSM - Qualitative Research in Health](https://www.sciencedirect.com/journal/ssm-qualitative-research-in-health), [Volume 2](https://www.sciencedirect.com/journal/ssm-qualitative-research-in-health/vol/2/suppl/C), December 2022, 100089

Section 3.1 of the results descibes the characteristics of the respondents. It might be helpful for your readers who don't know Bangladesh to define a registrar, and officer on special duty.

Response to Reviewer:

OSD: Being made an officer on special duty or OSD, as it is commonly known, is not something a newly appointed government official should be happy about.

The post is usually given to someone either as a punishment for corruption or irregularities or on political grounds. An OSD is an officer with no specific task assigned to him^[5]^.

Register doctor: Senior-Mid level positions of physicians with a clinical specialty.

We have now added footnotes to clarify these definitions.

Section 3. 2: can you explain how social networks are distinct from locally influential people? (I can guess but better to be explicit!)

Response to Reviewer: Our qualitative study revealed that the doctors who were working in their own area and who also had connections with local politicians and influential people coped easily in their rural posts. Some of the doctors built and maintained a good rapport with local people from the very beginning of their rural posting. They also maintained good relations with the local authorities, including the police and local government administration. These doctors indicated that monthly sub-district meetings that involved all significant stakeholders in the area were a very useful platform to discuss issues and to network. Some respondents also used their relationship with colleagues as a coping mechanism in rural settings. In the face of staff shortages, the remaining doctors managed their excess workload through mutual understanding^[6]^.

It would be helpful if Section 3.3 had a short table summarizing key findings.
Response to Reviewer: Thanks for your suggestions. We have already presented the findings in table 4.

Section 4 (discussion) refers to scarce logistics, pressure from local elites and political hoodlums for undue advantage... these were not explored in the results (or I missed them). How do these relate conceputally to attacks from the community, social networks, and political connections?

Response to Reviewer: Thanks for your feedback. We have revised this line to reflect our findings.

1. https://www.dhakatribune.com/bangladesh/nation/166696/acc-40%25-doctors-absent-in-workplaces
2. https://old.dghs.gov.bd/index.php/en/home/84-english-root/ehealth-eservice/103-office-attendance-monitoring-system
3. https://www.dhakatribune.com/bangladesh/nation/166696/acc-40%25-doctors-absent-in-workplaces
4. https://www.thedailystar.net/health/healthcare/news/absenteeism-headache-health-services-3120321
5. https://www.thedailystar.net/recruited-1-647-docs-lobby-to-be-osds-40116
6. https://ace.soas.ac.uk/wp-content/uploads/2022/05/WP29-WorkingPaper-Bangladesh-201015-1.pdf
