# Supplementary material for: Who is absent and why? Factors affecting doctor absenteeism in Bangladesh
Source: PLOS Glob Public Health. 2024 Apr 4;4(4):e0003040. doi: 10.1371/journal.pgph.0003040 (PMC10994372; doi:10.1371/journal.pgph.0003040)
Supplement: Attachment — Submitted filename: Response to reviewers comments 2 Bangladesh ACE PGPH.docx [file pgph.0003040.s003.docx]

Please find below a point by point response to the editorial and reviewer suggestions below with our response presented in blue.

**Editorial comments**

*Thank you for submitting this revision, and for your patience with the review process. The reviewer has provided a detailed review of this revision and finds that there continue to be several areas that require attention. You are invited to submit a revision that considers these comments carefully, particularly around methods and statistical analysis.*

Thank you, please see below for our response to each point raised and we have incorporated suggestions into the revised manuscript as described.

**Reviewer #1**

*The revised version of the manuscript shows improvement; however, there are still significant issues, particularly in the methods (sampling and analysis) and results (Tables 3, 4, 5 contain major errors, which are not acceptable at this stage of revision) sections. While the discussion section has improved from the earlier version, it does not fully align with the provided guidance, and some crucial explanations are still missing. Additionally, persistent formatting and syntax errors are present throughout the manuscript, which is not acceptable at this stage.*

Thanks again for your thorough review of our work. We have revised the discussion further and undertaken a thorough review of the syntax and formatting. In these ways we believe we have incorporated your suggestions into this version of the paper as described below.

***Abstract:*** *“Those who did not….”: The sentence reads confusing with a comma after "Those who did not."*

We have amended this sentence. See page 2 of manuscript:

*The main reasons for absenteeism reported by those who did not serve their term of rural posting uninterrupted were formal training opportunities (65%), family commitments (41%), and others (17%).*

***Keywords:*** *Why is Network written with a capital N? There are inconsistent capitalization in other keywords too.*
Thanks, we have revised the capitalization of the keywords. See page 2:

*Keywords: Doctor absenteeism, Accountability, Socio-political network, Bangladesh, Anti-corruption*

***Introduction:*** *There incorrect formatting of in-text citations, notably, 1, 2, 3.*

We have followed the PLOS Global Public Health Guidelines for in-text citations and will happily work with the journal to correct any issues if the work is ultimately accepted.

*“It also increases…and remote areas”: Very long sentence. Break it into two.*

We have broken this sentence into two as suggested . See page 3:

*Absenteeism which, for the purposes of this paper that focuses on rural facilities, have defined as leaving a rural posting early, for any reason, reduces the effectiveness of healthcare investments, compromises quality of services, and disproportionately affects vulnerable communities (4). It increases the burden on present health workers, decreasing their motivation and performance (5,6). Absenteeism also compounds existing system challenges relating to health workers, including shortages, maldistribution and poor retention, especially in rural and remote areas (7).*

“An increasing body … contexts they face.”: Please simplify this sentence.

We have simplified the sentence as follows:

*Increasingly, experts have argued that these measures have been ineffective because they do not address the key incentives driving the behaviour of doctors nor account for the different contexts they face (1,2,21,22).*

“found that doctors … places to work.”: Incomplete sentence.

Thanks for your suggestion. This line is now revised as follows:

There is evidence that multiple factors influence doctors’ availability in rural and remote public health facilities (23).

“Specifically, we defined … for any reason.”: Definition of absenteeism must be clarified much earlier; not as the last sentence of the background section. Shift this sentence to the place where you first introduced the term 'absenteeism.'

We have now moved it into the second sentence of the Introduction, as follows:

*Absenteeism, which for the purposes of this paper that focuses on rural; facilities, have defined as leaving a rural posting early, for any reason, reduces the effectiveness of healthcare investments, compromises quality of services, and disproportionately affects vulnerable communities (4).*

Overall comment: The writing style and sentence structure must be further tightened up. Please consider getting the manuscript thoroughly reviewed for language by a native English speaker.

We have reviewed throughout.

*Materials and methods:
Please add a section on sampling, including sample size calculation and sampling approach*.

Thank you for this comment and we apologise that we overlooked these details previously. We have added the following section:

***Sample***

*The survey was conducted in 2019 amongst doctors working at four tertiary hospitals who also had experience in rural facilities within the previous ten years. The facilities were chosen strategically to maximise recruitment given the budget available for data collection. The target sample size for the study was calculated using the following formula:*

$$n= \frac{Z_{1-\frac{\alpha}{2}}^{2}\times p\times(1-p)}{d^{2}},$$

*where n is the target sample size,* $Z$ *is the standard normal distribution (1.96),* $\alpha$ *is the level of significance (0.05), p is the population (assumed to be 0.50 since this would provide the maximum sample size)^[1]^. This gave a sample size of 384. The sampling strategy of this study was in line with the target sample size for the Discrete Choice Experiment survey (1). While there was no precise power calculation for the DCEs, target sample size of this study was 300. After the survey, 308 doctors were included in the final analysis. Doctors were enrolled in the survey following convenience sampling.*

*Analysis:
Please consult with a statistician to confirm if the application of a logistic regression analysis is appropriate in this context. This is particularly important as the authors did not clarify the sampling procedure.*

Thanks for your feedback. Logistic regression is a widely used regression model for modeling the probability of a binary outcome. Basic assumptions that must be met for logistic regression include independence of errors, linearity in the logit for continuous variables, absence of multicollinearity, and lack of strongly influential outliers^[2]^. We have checked the assumptions, and we are confident that this model is correctly applied. We have responded in more detail to each of your concerns below:

*Some important assumptions of logistic regression might have been violated:
1. Independence of Observations: The observations in the dataset are independent of each other. This assumption might have been violated if multiple doctors were surveyed from the same health facility.*

Although multiple observations were made in the same facilities, which could potentially give rise to concern, please note that we have modelled their past experience (factors) in the regression model. Also, it is very unlikely that they would be posted to the same rural facility. Consequently, we do not believe that the independence of observation assumption has been violated here.

2. Absence of multicollinearity: Nothing has been mentioned in the methods section if this has been checked.
Thanks for your suggestion. We have included the multicollinearity test in the manuscript. See page 7:

*To detect multicollinearity in the independent variables, the variance inflation factor (VIF) was tested. All the covariates showed VIF value less than three, so there was no indication of multicollinearity.*

*3. No regression diagnostics have been reported in the manuscript.*

We have included several regression diagnostics to ensure that the model is appropriate for the data and that the results can be trusted^[3]^. Results from a link test and Hosmer-lameshow's goodness-of-fit test reveal no misspecification in the model and our model fits the data well. This is reported on page 6:

*The adequacy and goodness-of-fit of the final regression model were diagnosed with link test, Hosemer-lameshow’s goodness-of-fit test.* *Results from the diagnostic tests reveal no misspecification in the model and the regression model fitted the data.*

*Optional: One suggestion for improvement is to elaborate on the rationale for choosing the 0.25 threshold for variable inclusion in the final model. Providing a brief justification could enhance the transparency of this decision.*

We agree with your feedback. We have now provided a brief justification and reference. See page 6-7:

*The final regression model included variables with p-values less than 0.25 in the crude model. To simplify the model and avoid multicollinearity, this selection strategy was followed.*

*General characteristics:
“Around 26% said … family commitments (41%).”: What does ‘uninterrupted mean here? Few days of interruption, few weeks, months, not completing the full 2 years? Please provide an operation definition in the methods section.*

and

*“Almost all respondents … staff being absent (50%).”: What does staff mean here? Another physician, a nurse, any stuff? Please specify. Provide an operational definition in the methods section.*

Added. See:

*2.6 Operational definitions*

*‘Absenteeism’ is defined as the doctors leaving their rural posting early, for any reason whereas ‘uninterrupted service’ means that doctors are fulfilling their rural postings without taking any breaks or leaving the assigned area without having obtained official permission. We have defined ‘staff’ as doctors, nurses, and those in supporting roles who work in the hospital. 'Political connections' refer to any affiliations, associations, or relationships that individuals may have with local politicians or high-level government officials. These connections could encompass personal relationships, affiliations with political parties or interest groups, or any form of interaction or influence that individuals have with those in positions of political power.*

*“To mitigate these challenges…”: there is a full stop after ‘neighbours’. Please correct it.*

This is now revised.

*“At the same time … rural postings.”: This statement is not clear. They did not get a chance because they were not allowed to do private practice, or they did not find time for private practice due to other commitments such as higher education, family, etc.? Please write the results accordingly to what you actually asked in the questionnaire.*

We asked whether they engaged in private practice during their rural posting or not. We have now revised the sentence per our questionnaire. See:

*At the same time, around 30% of the respondents said they were not involved in private practice during their rural postings.*

*Table 2: “Served each period of rural posting uninterruptedly”: What does the 'each period of rural posting' mean? Why not just ask 'Served rural posting uninterruptedly?*

Respondents may have served in different rural postings for different periods of time. That’s why we mentioned each period of rural posting separately rather than grouping them in a period of multiple rural postings.

*Table 2, under ‘Workload in the rural facility’: What is the difference between the response categories ‘Usually too much to be manageable’ and Not manageable’? In my opinion, they don’t necessary impart any meaningful difference. If the authors agree, they should recode the response categories and re-run the analysis*.

On reflection we agree with this point and have recoded the response categories as per your suggestion.

*Table 3: Why do these percentages not add up to 100? I understand these are chi-squared tables, but this way of presenting the tables is confusing and does not convey a clear meaning. Please revisit this table.*

Originally this table reported estimates for doctors who served in a rural posting without interruption. Thus, only one cell from the bivariate table was reported. We have now revised our analysis based on your thorough review and have included all categories such that the percentages add to 100 for every category.

*Table 4: Same comment as Table 3. If you are trying to present your chi-squared test findings in a tabulated form, please use an appropriate table format. Perhaps you want to show only the significant association, i.e., the association between uninterrupted rural stay and personal network.*

We have now revised this table as described above.

*Table 5: This table has multiple mistakes:
1. Networking variables are not identified like career progression and work experience variables.
2. Network variables are wrongly identified as career progression variables.
3. Model 2 includes network variables too, as evidenced by the table, but the text does not say so. The text says that model 2 includes rural experience and socio-demographic variables.*

As described below, we have now revised the regression analysis as per your suggestion. Thanks for your feedback. These issues are resolved now.

*Overall comment: I am unsure what additional value the complicated three-model analysis adds as opposed to a single full model. The significant variables in models 2 and 3 are the same. The only additional significant variable in model 1 is age, which may not be too intriguing. So, instead of three models, a single full model and an unadjusted model might be useful and easier to comprehend.*

We have now revised our analysis and only presented crude odds ratio and adjusted odds ratio in table 5. Thank you for your suggestion.

*Discussion and conclusions:
In my earlier review, I suggested presenting the major findings directly and declaratively in the first paragraph of the discussion section. This has not been respected. I am repeating my suggestion again for improving the manuscript in its future iteration:
1. State the study's major findings in the first paragraph in a direct, declarative and succinct manner. You may select two or, at best, three major findings from your study.*

We have revised the opening paragraph of the discussion as follows:

*About one-quarter of the cohort of doctors in this study with recent experience working in rural Bangladesh reported leaving their rural posting early. Doctors reported facing significant challenges working in rural areas, including feeling physically unsafe, experiencing verbal and physical abuse, staff shortages, non-cooperative colleagues, theft and poor-quality housing.* *Doctors who reported being well-supported by the community through access to influential people were more than twice as likely as others to complete their rural posting. While some previous literature has highlighted the challenging conditions that these doctors face, there have been little quantitative data published on the type and extent of these challenges. These results provide a compelling (albeit partial) explanation for the historical difficulties observed in overcoming absenteeism in these facilities as well as the broader issues of attracting and retaining doctors to serve rural areas in Bangladesh.*

*2. Write one paragraph or two for each major finding by explaining the meaning and importance of the findings.*

We have followed this structure.

*3. Relate the findings to those of similar findings, preferably from Bangladesh or similar settings.*

We have related each finding to the relevant literature.

*4. Mention the limitation and strengths of the study in a separate paragraph.*

Done. See:

*Our research was subject to several limitations. Resource constraints limited our sample to doctors working in four large urban hospitals with recent experience of working at rural facilities, and thus may not be generalisable to the broader medical population servicing rural communities. Given the sensitivity of the topic of absenteeism in Bangladesh, respondents may not have been comfortable being entirely open when responding to some of the questions. We attempted to minimise the impact of such bias through an extensive survey development process, including qualitative work and piloting with the target population. Despite these steps, recruitment was challenging and we fell short of our target sample size. Nonetheless, the significance and fit of our models suggest that we have identified significant relationships between our variables of interest and absenteeism of doctors at rural postings in Bangladesh.*

*5. Provide your opinion about future research and/or policy implications, research/policy recommendations. Do it in a separate paragraph instead of clubbing them with each paragraph.*

See:

*Ultimately, our work suggests that the experience junior doctors have when posted to a rural facility is likely to be challenging but will vary widely depending on how safe and supported they feel. Addressing these challenges should be a priority and these findings have important implications for policies seeking to overcome doctor absenteeism in Bangladesh by providing a unique insight to doctors’ perspectives to a problem that has largely been neglected, aside from a few related studies (1,27,33). While attention has traditionally focused on increased monitoring of doctors and enforcement of regulations (for example, punishing rule breakers), our results build on existing literature and suggest that it may be more effective to focus attention and scarce health system resources on improving the working conditions facing doctors who wish to be present at work. Forging strong linkages with local communities so that providers receive support and protection from violence appears to be a promising channel for further investment^[4]^. Further, allowing for flexibility in circumstances where doctors have opportunities to attend training courses to improve their skills without being penalised will likely be highly valued by doctors and could improve their retention and motivation to serve in rural communities.*

*6. In the conclusion section or the last paragraph of the discussion section, give a take-home message, i.e., a couple of sentences summarizing your research and things you want the reader to remember, at the least from your article.*

See:

*Despite these limitations, this work represents a unique contribution to the literature by providing empirical data from the perspective of doctors in Bangladesh, a perspective that is surprisingly overlooked in debates about doctor absenteeism. Our findings build on recent developments in the field suggesting that it may be more pragmatic and cost-effective to focus on meeting the needs of doctors who can be induced to attend work rather than monitoring and punishing offenders.*

“We found that … rural posting.”: This indicates that doctors with local support are more likely to stay in rural areas. This also means that the tendency of doctors to local stay is contingent on informal local support. This eventually means there is a lack of formal support system for the doctors to encourage and support them to stay there. These can be supported by other literature from Bangladesh and similar settings.

*We agree with you. We have described this point in response to the point 5 about the discussion above with relevant literature.*

*Also, delete the extra full stop from the sentence.*

*Deleted*

*Also, this paragraph does not attempt to substantiate the discussions with findings from Bangladesh or similar contexts.*

“It is also doubly … training opportunities.”: Instead of this, write, “It also doubly penalise doctors in the rural areas as they are both more likely to be overworked and the absence of some of their colleagues adds to the existing high workload.”

We have revised this sentence to read: *It also doubly penalises doctors in the rural areas as they are both more likely to be overworked and less likely to access such training opportunities. This experience mirrors that in other nations (29–31).*

“However, this study … rural posting period.”: It is possible that their private practice is not in their area of rural posting but rather in a different, perhaps urban, area. This finding and the associated paragraph deserves more thoughtful explanations.

*We have now elaborated our findings. Please see page 14:*

*There is a common belief that the doctors remaining in the rural postings are compensated by flourishing private practice, an opportunity to be with the family, or a residence near the workplace provided by the authority (23,32). However, this study did not find a significant relationship between private practice (almost 30% of our sample) and completing the rural posting period.*

This sentence should be read in conjunction with the previous one – *“There is a common belief that the doctors remaining in the rural postings are compensated by flourishing private practice, an opportunity to be with the family, or a residence near the workplace provided by the authority (23,32).”* Because we were asking about private practice in general, it would capture work undertaken in urban and rural areas. We are not sure what we can add here.

“Policies around training … education or training.”: This part of the paragraph is discrete from the previous arguments. Please create a separate paragraph.

We have moved this to the previous paragraph:

*This study also heard reports that doctors in rural areas have to leave their posts frequently to complete official training (short or long term) or higher studies sponsored by the authorities. Such schemes operate against efforts to secure a stable health workforce for the rural population. It can take a long time for lost doctors to be replaced, given complex administrative procedures and the shortage of doctors to fill the newly created vacancies (27,28). It also doubly penalises doctors in the rural areas as they are both more likely to be overworked and less likely to access such training opportunities. This experience mirrors that in other nations (29–31). Policies on training need to be carefully calibrated such that they do not create a situation whereby doctors are incentivised not to complete postings in rural communities. This view is supported by results from a discrete choice experiment in this population that point to the value of prioritizing doctors with a good attendance record through provision ‘bonus points’ that enable preferential placement in higher education or training (1).*

“There are several limitations … target population.”: In my opinion, the most important limitation is the nonprobability sampling method of your study, although the authors did not mention anything about the sampling procedure.

*Our research was subject to several limitations. Resource constraints limited our sample to doctors working in four large urban hospitals with recent experience of working at rural facilities, and thus may not be generalisable to the broader medical population servicing rural communities. Given the sensitivity of the topic of absenteeism in Bangladesh, respondents may not have been comfortable being entirely open when responding to some of the questions. We attempted to minimise the impact of such bias through an extensive survey development process, including qualitative work and piloting with the target population. Despite these steps, recruitment was challenging and we fell short of our target sample size. Nonetheless, the significance and fit of our models suggest that we have identified significant relationships between our variables of interest and absenteeism of doctors at rural postings in Bangladesh.*

1. Daniel, W. W. (1978). *Biostatistics: a foundation for analysis in the health sciences*. Wiley.
2. Stoltzfus, J. C. (2011). Logistic regression: a brief primer. Academic emergency medicine, 18(10), 1099-1104.
3. Long, J. S., & Freese, J. (2006). *Regression models for categorical dependent variables using Stata* (Vol. 7). Stata press.
4. Cutchin, M. P. (1997). Physician retention in rural communities: the perspective of experiential place integration. *Health & Place*, *3*(1), 25-41.
